# Early Life Stress Inhibits Expression of Ribosomal RNA in the Developing Hippocampus

**DOI:** 10.1371/journal.pone.0115283

**Published:** 2014-12-17

**Authors:** Lan Wei, Jin Hao, Arie Kaffman

**Affiliations:** Department of Psychiatry, Yale University School of Medicine, New Haven, Connecticut, United States of America; Shanghai Jiaotong University School of Medicine, China

## Abstract

Children that are exposed to abuse or neglect show abnormal hippocampal function. However, the developmental mechanisms by which early life stress (ELS) impairs normal hippocampal development have not been elucidated. Here we propose that exposure to ELS blunts normal hippocampal growth by inhibiting the availability of ribosomal RNA (rRNA). In support of this hypothesis, we show that the normal mouse hippocampus undergoes a growth-spurt during the second week of life, followed by a gradual decrease in DNA and RNA content that persists into adulthood. This developmental pattern is associated with accelerated ribosomal RNA (rRNA) synthesis during the second week of life, followed by a gradual decline in rRNA levels that continue into adulthood. Levels of DNA methylation at the ribosomal DNA (rDNA) promoter are lower during the second week of life compared to earlier development or adulthood. Exposure to brief daily separation (BDS), a mouse model of early life stress, increased DNA methylation at the ribosomal DNA promoter, decreased rRNA levels, and blunted hippocampal growth during the second week of life. Exposure to acute (3 hrs) maternal separation decreased rRNA and increased DNA methylation at the rDNA proximal promoter, suggesting that exposure to stress early in life can rapidly regulate the availability of rRNA levels in the developing hippocampus. Given the critical role that rRNA plays in supporting normal growth and development, these findings suggest a novel molecular mechanism to explain how stress early in life impairs hippocampus development in the mouse.

## Introduction

A growing body of work has demonstrated abnormal hippocampal development in children exposed to abuse or neglect [1]–[5]. Early life stress (ELS) modifies hippocampus development across diverse mammalian species, including rodents, suggesting that animal models may help elucidate the molecular and cellular changes that guide these developmental changes in children [6], [7]. Unfortunately, most of the animal work to date has focused on the ability of ELS to modify hippocampal structure and function in adulthood, with little effort made to systematically study how ELS modifies hippocampal development [8]. To address this issue, we developed a mouse model of ELS called brief daily separation (BDS). In this model, pups of a highly stress-reactive strain (BALB/cByj) are raised in the absence of nesting material to allow for careful maternal observation and to mimic impoverished conditions. During the first three weeks of life, half of the litters are separated from their dam for 15 min daily (BDS condition), while the remaining litters are left undisturbed (control condition). Exposure to BDS increases corticosterone levels, blunts the normal growth-spurt seen in the hippocampus at around PND14 [9], and is associated with impaired hippocampal-dependent memory in adulthood [8].

The molecular mechanisms by which BDS inhibits hippocampal growth have not yet been identified. Here we tested the hypothesis that BDS impairs hippocampal growth by inhibiting rRNA synthesis during a critical period in hippocampus development. Several reasons guided our decision to examine this issue. First, the hippocampus undergoes an accelerated growth phase during the second week of life [9], and rRNA synthesis is essential to support cellular growth and differentiation [10]. Second, the rate of rRNA synthesis is highly regulated by a host of environmental factors including stress [10]. For example, incubating a lympho-sarcoma cell line with 10 µM of Dexamethasone causes cell cycle arrest by inhibiting rRNA synthesis [11]. Third, blocking rRNA synthesis in neural stem cells (NSC) causes rapid cell-cycle arrest and apoptosis [12]. This issue is particularly pertinent given the large wave of NSC proliferation taking place in the hippocampus during the first 2 weeks of life [13] and the observation that BDS decreases DNA content in the developing hippocampus [9]. Similarly, transient inhibition of protein synthesis in 7-day old pups, with cyclohexamide, blocks NSC proliferation, decreases DNA content in several brain regions and causes behavioral changes that persist into adulthood. Therefore, inhibition of rRNA synthesis could provide a powerful molecular switch by which ELS impairs hippocampal growth.

In support of this hypothesis, we show that the rate of rRNA synthesis in the normally developing mouse hippocampus peaks during the second week of life. This peak in rRNA levels coincides with rapid hippocampus growth, and is associated with a decrease in DNA methylation at promoter elements that have been shown to regulate rRNA transcription [14]. We found that exposure to BDS increased DNA methylation at the rDNA promoter in a manner that was highly correlated with its ability to inhibit rRNA levels in 14-day old pups. Decreased rRNA synthesis at PND14 was associated with decreased DNA content in the hippocampus of PND14 and PND28 offspring, suggesting that inhibition of rRNA during the second week of life blunts hippocampal growth. Finally, exposure to a single 3 hr period of maternal separation, in 14-day old pups, was sufficient to decrease rRNA and to increase DNA methylation at the rDNA promoter. These results indicate that stress early in life causes rapid changes at promoter elements that regulate rRNA synthesis. Together, this work suggests a novel molecular mechanism to explain how stress early in life modifies hippocampal development in the mouse.

## Materials and Methods

### Animals

Mice were housed in standard Plexiglas cages and maintained on a standard 12:12 hr light-dark cycle (lights on at 7:00AM), under constant temperature and humidity (22 °C and 50%), and with food provided *ad libitum*. All the animal work was done with BALB/cByj mice (Stock # 001026, Jackson Laboratories).

### Brief daily maternal separation (BDS)

Sixty female and 30 male BALB/cByj mice (6–8 weeks old) were purchased from Jackson Laboratory and allowed to acclimate for two weeks. Mating and BDS exposure were done as previously described [9]. In brief, 2:1 female-to-male breeding harems were set up, and visibly pregnant dams were individually housed in maternity cages in the absence of nesting material. At birth (PND = 0), pups were culled to 6–8 pups per litter, and litters were randomly assigned to either brief daily separation (BDS) or control groups. The separation procedure occurred daily between 11:00–11:40AM from PND1-21. During each BDS session, the dam was removed from the home cage, placed in a holding cage covered with fresh corncob bedding, and provided with food and water. Pups were transferred individually and placed at different corners of a new standard cage (20.3 × 27.9 cm) covered with clean corncob bedding. Cages were left undisturbed for 15 min at ambient temperature in the vivarium (i.e. 22°C ±2°C), after which pups were transferred back to their home cage, followed by the dam. On PND 22, pups were weighed and housed in groups of 3–5 littermates of the same sex.

### Developmental studies

Forty female and 20 male BALB/cByj mice (6–8 weeks old) were purchased from Jackson Laboratory and allowed to acclimate for two weeks. To assess normal hippocampal development, all mice were subjected to the control condition outlined above (no BDS group). Brain tissue was harvested at six developmental time points: PND 7, 14, 22, 28, 42, and 63. Mice were rapidly decapitated and the hippocampi were dissected rapidly and frozen in liquid nitrogen. Tissue from 5–6 litters was collected for each age group. All dissections were done between 2:00–5:00 PM to minimize circadian effects on gene expression. Hippocampal tissue (n =  9 males per age group) was rapidly thawed in cold lysis buffer from the AllPrep DNA/RNA/Protein mini kit (Cat# 80004, Qiagen), homogenized (10 × 1 sec pulses) using an ultrasonic processor (Cole Parmer CP70), and spun at 16,000 g for 3 min. Small aliquots of the homogenates were used to quantify total levels of DNA and RNA in the hippocampus using a Qubit Fluorometer (Life Technologies), as previously described [8], [9]. RNA, DNA and proteins were then purified using the AllPrep DNA/RNA/Protein mini kit according to manufacturer instructions. Purified RNA and DNA were quantified using a NanoDrop 2000 (Thermo scientific).

### Quantitative PCR (q-PCR)

cDNA was synthesized using 500 ng of total RNA and random hexamer primers, using the SuperScript III RT kit (Invitrogen). Q-PCR was performed using Quantitact-SyBr Green and analyzed on a 96-well, Stratagene Mx3000 thermocycler. Ribosomal RNA levels in the hippocampus were assessed using 18s-rRNA-2F: GCATGCACTCTCCCGTTC and 18s-rRNA-2R: AGCGCGAGAGAGGAGGAG primers that target the 18S rRNA subunit. TBP-F-1: AAAGGGAGAATCATGGACCAGAACAA and TBP-R-1: TGGACTAAAGATGGGAATTCCAGGAG primers were used to assess levels of the TATA binding protein (TBP), which was used as an internal control. rRNA-2F: AGTTGTTCCTTTGAGGTCCGGTTCTT containing a HpaII site (underlined) at CG_6 (−143) and rRNA-2R: AAAGAGACAGGGAGGAAAGTGACAGG were used to amplify a 113 bp amplicon of the rDNA promoter. Efficiency of all primers was confirmed to be 100% +/−10% and the 2^-ΔΔCT^ method was used [15] to calculate relative RNA levels. The calibrator groups used in different experiments are specified in the figure legends.

### Bisulfite treatment and pyrosequencing

Genomic DNA was harvested from the hippocampus using the DNA/RNA/Protein mini kit as described above. Three hundred nanograms of genomic DNA were bisulfite treated with the EZ DNA Methylation-Gold Kit (Zymo Research, Cat# D 5005) according to manufacturer instruction. The rDNA proximal promoter was subsequently PCR amplified and pyrosequenced using a Mouse Rn45s assay #ADS3841 developed for this purpose by EpigenDx (Hopkinton, MA 01748). A 251 bp amplicon containing the rDNA proximal promoter was PCR amplified using the ADS3841FP and the biotinylated primer ADS3841RPB, and was shipped for Pyrosequencing at EpigenDx. A total of 6 µL containing 2 µL of PCR reaction and 4 µL of ddH_2_0 were loaded into a PSQ 96HS sequencing machine and sequenced using the ADS3841-FS1 primer. One technical detail regarding this assay is that site CG_5 has shown decreased DNA methylation compared to all other sites most likely due to a heterogeneous G/T SNP that is found close to this site. This sequence heterogeneity may interfere with correct assessment of the methylation rate at this site. Additional details regarding the Mouse Rn45s assay (cat #ADS3841) are available at EpigenDx.

### HpaII methylation assay

HpaII digestion or sham digestion were performed in triplicates using 50 ng of genomic DNA per reaction digested with either 10 U of HpaII (NEB, Cat# R0171L) or no HpaII (sham digestion), for 2 hrs at 37°C, followed by heat inactivation at 80°C for 20 min. Reactions were then spun briefly, and levels of intact DNA were assessed with q-PCR using rRNA-2F and rRNA-2R primers. Note that these primers amplify a 113 bp fragment containing a single HpaII site at CG_6 and that this site is cleaved by HapII only if it is not methylated. The % methylation at CG_6 for each animal was calculated by first determining the average CT for the three sham digested reaction (CT _sham ave_), which was then used to calculate the *delta CT_rxn_* =  CT_HpaII_ – CT _sham ave_ for each individual reaction. Second, the *delta CT_rxn_* for the three triplicates was averaged to obtain the delta CT per animal (delta CT). Third, the % DNA methylation at CG_6, for each animal, was calculated as  =  (1/2^delta CT^) *100.

### Acute maternal separation (AMS)

Acute maternal separation was done as described previously [8]. Fourteen-day old pups raised under control conditions (see above) were either kept undisturbed with the dam (control), or were placed individually in a new cage (AMS) with clean bedding and maintained at ambient temperature in the vivarium (i.e. 22± 2¡C). After 3 hrs of maternal separation, male pups were rapidly sacrificed to collect plasma and to harvest hippocampal tissue, as described previously [8], [9].

### Plasma corticosterone levels

Corticosterone levels were assessed using the Corticosterone ELISA kit (Enzo Lifesciences 96-well assay kit, cat # ADI-900-097), with 10 µL of plasma ran in duplicates for each sample, according to manual instructions.

### Statistical analysis

Details regarding the statistical analyses used can be found in the figure legends. The data were carefully screened for inaccuracies, outliers, normality, homogeneity of variance, and sphericity. The SPSS Statistics 19 software (IBM) was used for statistical analysis with *p*<0.05 considered as significant.

### Ethics

All studies were approved by the Institutional Animal Care and Use Committee (IACUC) at Yale University and were conducted in accordance with the recommendations of the NIH Guide for the Care and the Use of Laboratory Animals.

## Results

### Levels of rRNA peak at PND14 in the developing hippocampus in a manner that correlates with reduced DNA methylation at the rDNA proximal promoter

**Figure 1:**
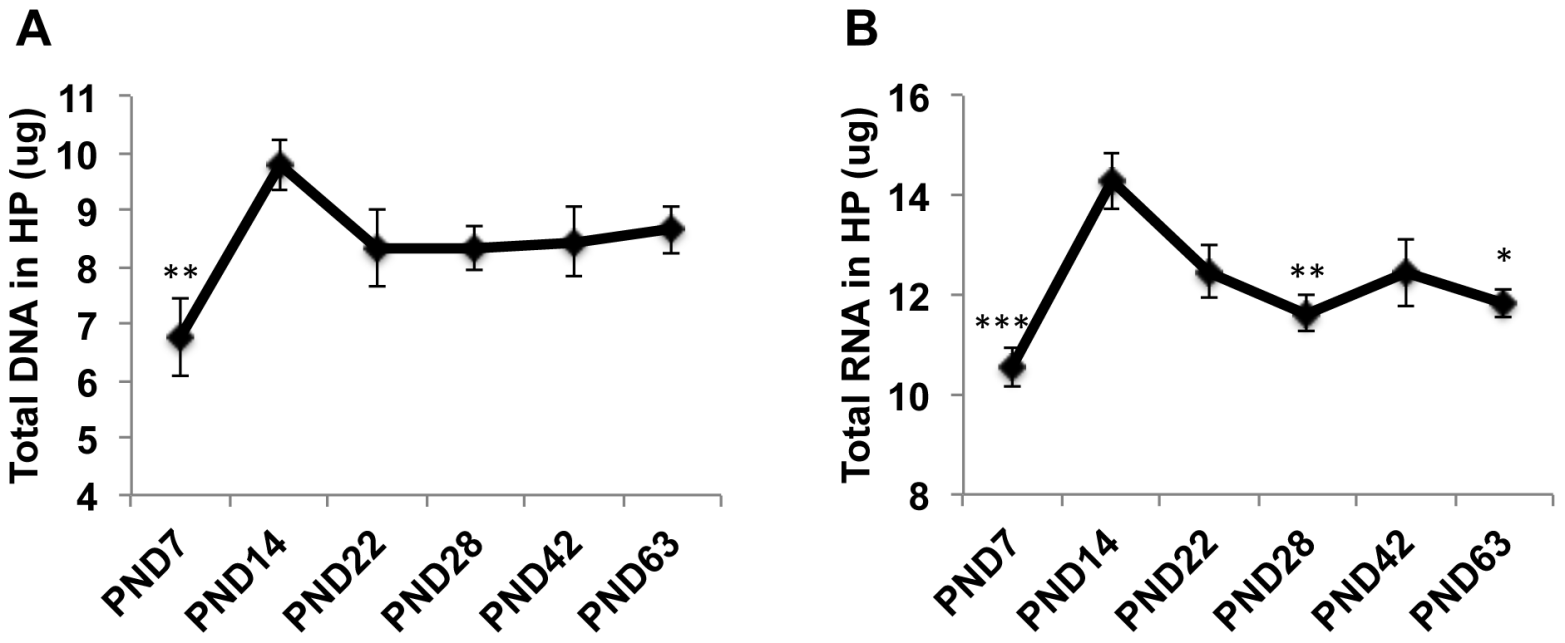
The developing hippocampus undergoes accelerate growth during the second week of life. Total levels of DNA (A) and RNA (B) extracted from the hippocampus at different developmental ages. N = 9 male mice per age. Error bars represent mean ± SEM. *p<0.05, **p<0.01, ***p<0.0005, One-way ANOVA followed by Tukey-post hoc analysis when compared to PND14.

**Figure 2:**
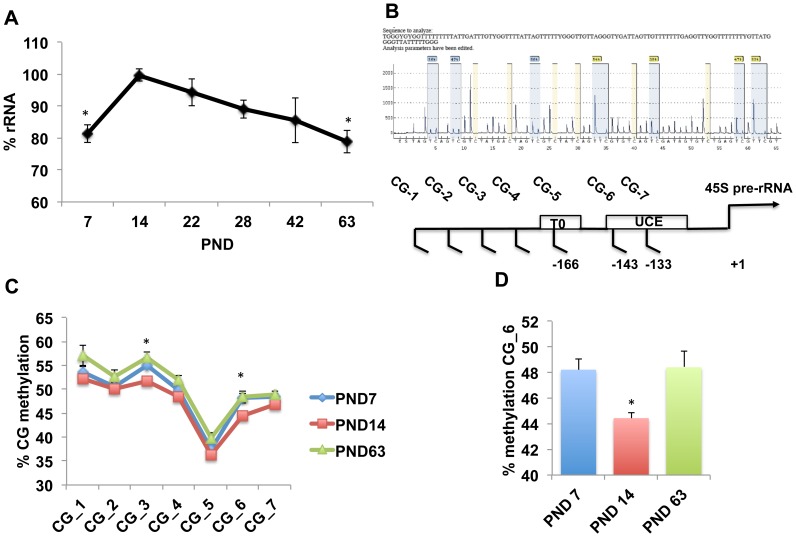
rRNA levels in the hippocampus peak during the second week of life in a manner that is associated with reduced DNA methylation at the rDNA promoter. A. rRNA levels peak during the second week of life. B. (top), a representative pyrogram of the pyrosequencing assay used to assess DNA methylation (blue) lines, (bottom) schematic diagram of the rDNA promoter elements. C. DNA methylation at the rDNA is lower in PND14 compared to PND7 and PND63 for CG_3 and CG_6. D. DNA methylation levels at CG_6 on PND7, 14, and 63. One-way ANOVA followed by Tukey-post hoc analysis when compared to PND14 (A, D), repeated measures ANOVA followed by simple-effect analysis (C). Error bars represent mean ± SEM, *p<0.05. UCE-upstream core element, To- Proximal-promoter terminator sequence.

Only male offspring were included in the study because our previous work [8], [9] and studies from other laboratories indicated that behavioral sequelae of ELS in rodents are more robust in male offspring [16]–[21]. Using an independent cohort of male offspring, we replicated our previous work [9] showing that the total levels of DNA in the hippocampus peak at around two weeks, F(5,48) = 3.11, p =  0.016 (Fig. 1A). Similar results were seen for total amount of RNA harvested from the hippocampus, F(5,48) = 13.71, p<0.0005 (Fig. 1B). After PND14, levels of DNA and RNA declined to levels seen in adulthood. These data indicate that the hippocampus undergoes a rapid growth spurt during the second week of life, followed by a pruning phase that extends into adulthood. Since a high rate of rRNA synthesis is required to support rapid developmental growth [10], we tested whether levels of rRNA in the hippocampus also increase during the second week of life. Indeed, levels of rRNA peaked at PND14 after which they decline linearly into adulthood, F(4,19) = 25.09, p<0.0005 (Fig. 2A). Note that rRNA levels at PND14 represent a true enrichment because the same amount of total RNA (500 ng) was used across all ages.

Next, we tested whether the increase in rRNA synthesis seen in 14-day old pups is associated with a transient decrease in DNA methylation at rDNA promoter sequences that were previously shown to regulate rRNA synthesis [14]. To accomplish this, we collaborated with EpigenDx (Hopkinton, MA 01748) to develop a pyrosequencing assay to determine levels of DNA methylation across 7 methylation sites within the rDNA proximal promoter (Fig. 2B). Using hippocampal DNA obtained from PND7, PND14 and PND63 male mice (n = 8–9 for each age group), we found an overall decrease in DNA methylation at PND14 compared to PND7 and PND63 mice, main effect of age, repeated measure ANOVA, F(2,21) = 4.162, p =  0.03, (Fig. 2C). Post-hoc analysis showed a significant decrease in DNA methylation at PND14 compared to PND7 and PND63 for CG_3 and CG_6 (Fig. 2C–D). The finding that DNA methylation at CG_6 decreases at PND14 (Fig. 2D) is particularly interesting given that the transcription factor UBF binds at this site [14]. These findings are the first to demonstrate that the rate of rRNA synthesis increases while DNA methylation at the rDNA promoter decreases during the second week of life in the hippocampus, and suggest that this process plays an important role in supporting the accelerated growth seen during this period in the hippocampus.

### Exposure to BDS decreases rRNA expression and increase DNA methylation at the rDNA proximal promoter

**Figure 3:**
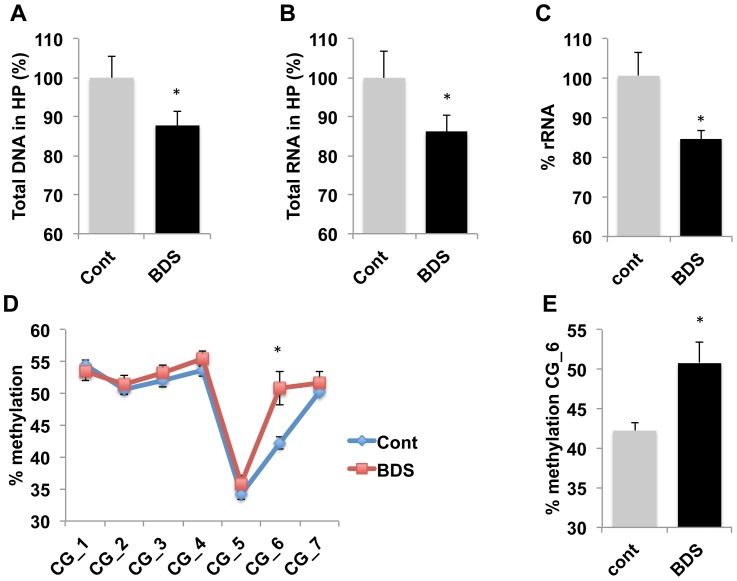
Exposure to BDS decreases rRNA levels and increases DNA methylation at the rDNA promoter of 14-day old pups. Exposure to BDS decreases total amount of DNA (A) and RNA (B) harvested from the hippocampus of 14-day old pups. C. Relative levels of rRNA are lower in BDS compared to control 14-d old pups. Exposure to BDS increases DNA methylation at CG_6 (D and E). Unpaired Student *t* tests (A, B, C, E), Repeated measures ANOVA followed by simple-effect analysis (D). Error bars represent mean ± SEM, *p<0.05.

Exposure to BDS decreased the total amount of DNA, t(27) = 1.89, p =  0.035, (Fig. 3A), and RNA, t(27) = 1.82, p =  0.039 (Fig. 3B), extracted from the hippocampus of PND14 pups. These findings replicate our previous work showing that BDS blunts the normal growth spurt seen in the hippocampus during the second week of life [9]. PND14 BDS pups also showed a significant decrease in rRNA levels in the hippocampus, t(10) =  2.53 p =  0.03, Fig. 3C. There was a significant inverse correlation between plasma corticosterone levels and rRNA levels (*r* =  −0.57, *p* = 0.034), suggesting that increased corticosterone levels seen in BDS pups are responsible for inhibiting rRNA synthesis in the developing hippocampus. Next, we tested whether exposure to BDS increased DNA methylation at the rDNA promoter. There was a significant interaction between methylation site and exposure to BDS, F(6,78) =  3.76, p =  0.035, but no significant main effect of BDS, F(1,13) =  2.82, p =  0.116 (Fig. 3D). Planned simple effect comparisons for CG_3 and CG_6 (see Fig. 2C) showed a significant increase in DNA methylation at CG_6 (Fig. 3E, F(1,13) = 6.98 p =  0.02), but not for CG_3, F(1,13) = 0.16 p =  0.69. This is the first study to show that exposure to ELS increases DNA methylation at the rDNA promoter and reduces rRNA availability in the developing hippocampus.

**Figure 4:**
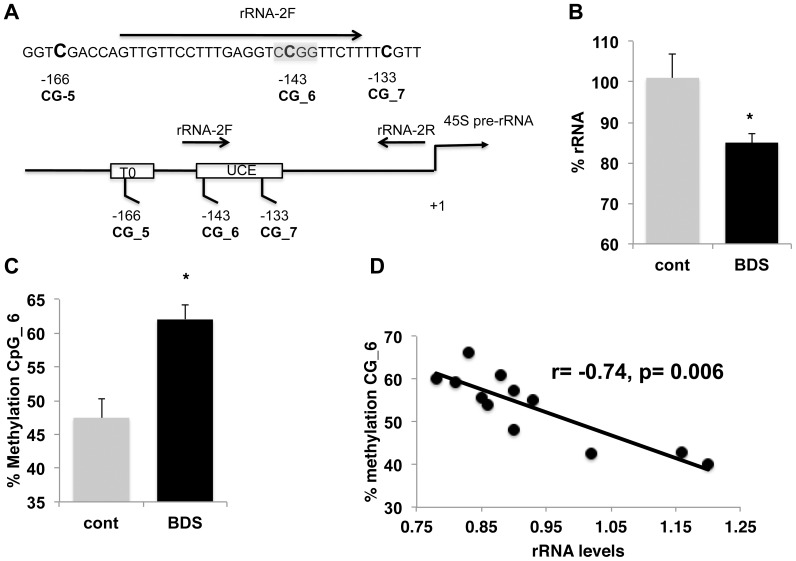
PND14 pups exposed to BDS show increased methylation rate at CG_6 in a manner that is highly correlated with a decrease in rRNA levels. A (top) Nucleotide sequence of primer rRNA-2F (arrow) is shown with HpaII restriction site at CG_6 is highlighted in grey, (bottom), schematic diagram of Q-PCR primers used to assess DNA methylation at CG_6. Note that no product will be generated if CG_6 is not methylated and the DNA is digested with HpaII. Exposure to BDS decreases rRNA levels (B) while increasing DNA methylation at CG_6 in 14-day old pups (C). D. Levels of methylation at CG_6 are inversely correlated with rRNA levels in the developing hippocampus. Unpaired Student *t* tests (B, C), Pearson correlation (D). Error bars represent mean ± SEM, *p<0.05.

To further solidify these findings, we took advantage of the fact that the CG_6 site contains a HpaII restriction site that can be used to assess site-specific DNA methylation (Fig. 4A). We harvested genomic DNA and rRNA from the hippocampus of a different cohort of 14-day old control and BDS pups. Using this assay (see materials and methods for more details) we confirmed that BDS decreased rRNA [Fig. 4B, %, t(10) =  2.53, p =  0.03] and caused a significant increase in DNA methylation at CG_6 [Fig. 4C, t(10) =  4.1, p =  0.002]. Moreover, we found a strong negative correlation between methylation levels at this site and rRNA levels (r =  −0.74, p =  0.006, Fig. 4D). These findings provide an independent method to demonstrate that exposure to BDS increases methylation rate at CG_6, while reducing rRNA availability in the developing hippocampus.

### The effects of BDS on rRNA levels and DNA methylation at the rRNA do not persist in 28-old offspring

**Figure 5:**
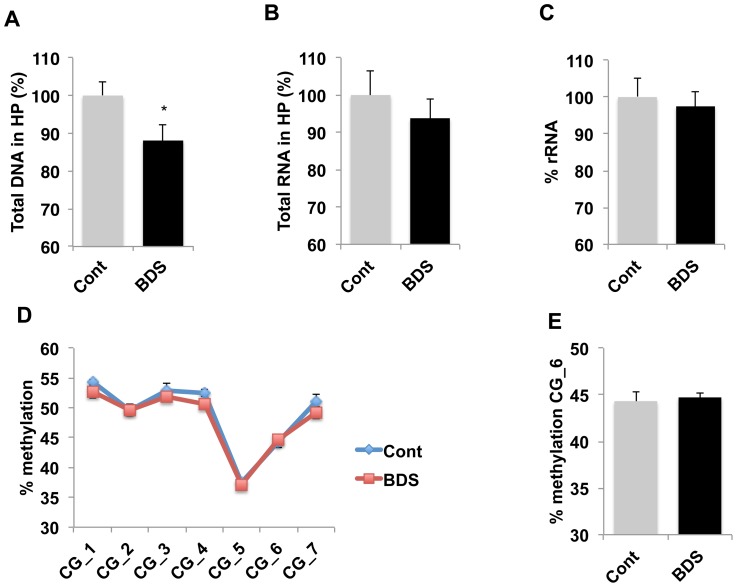
Exposure to BDS reduces DNA content in 28-day old pups without affecting levels of rRNA or DNA methylation at CG_6. Exposure to BDS decreases total amount of DNA (A), but not RNA (B), harvested from the hippocampus of 28-day old offspring. C. Relative levels of rRNA are similar in BDS compared to control 28-d old mice. The effects of BDS on DNA methylation at CG_6 do not persist in 28-day old mice (D and E). Unpaired Student *t* tests (A, B, C, E), Repeated measures ANOVA (D). Error bars represent mean ± SEM, *p<0.05.

Previous work has shown that some forms of early life stress are able to program DNA methylation of promoter elements in a manner that persists into adulthood [22]. These findings provided a mechanism by which events early in life are able to alter gene expression in the adult brain, and modify brain function and behavior in adulthood. Interestingly, suicide subjects exposed to abuse or neglect early in life showed a decrease in rRNA levels in the hippocampus that was associated with increased DNA methylation at the rDNA promoter [23]. These findings raised the possibility that stress early in life may regulate the availability of rRNA later in life by increasing methylation at the rDNA promoter. We therefore tested whether the effects of BDS on rRNA levels and DNA methylation at the rDNA promoter persisted in 28-day old juvenile offspring. This age was chosen because it allows offspring one week to recover from the acute effects of BDS, and because the DNA and RNA content in the hippocampus reach their adult size at this age (Fig. 1). Exposure to BDS caused a 15% reduction in DNA levels in the hippocampus, t(29) =  2.58, p =  0.039, Fig. 5A, but did not affect the total amount of RNA in the hippocampus (Fig. 5B). Levels of rRNA (Fig. 5C) and DNA methylation at the rDNA promoter (Fig. 5 D–E) were similar in PND28 BDS and control mice. These findings, suggest that the ability of BDS to regulate rRNA levels and DNA methylation at the rDNA promoter do not persist in the absence of ongoing stress. Nevertheless, the observation that the DNA content in the hippocampus was lower in BDS mice raises the possibility that transiently inhibiting rRNA during the second week of life inhibits neurogenesis during this critical period in manner that modifies hippocampus structure and function later in life.

### Acute maternal separation is sufficient to increase DNA methylation at CG_6

**Figure 6:**
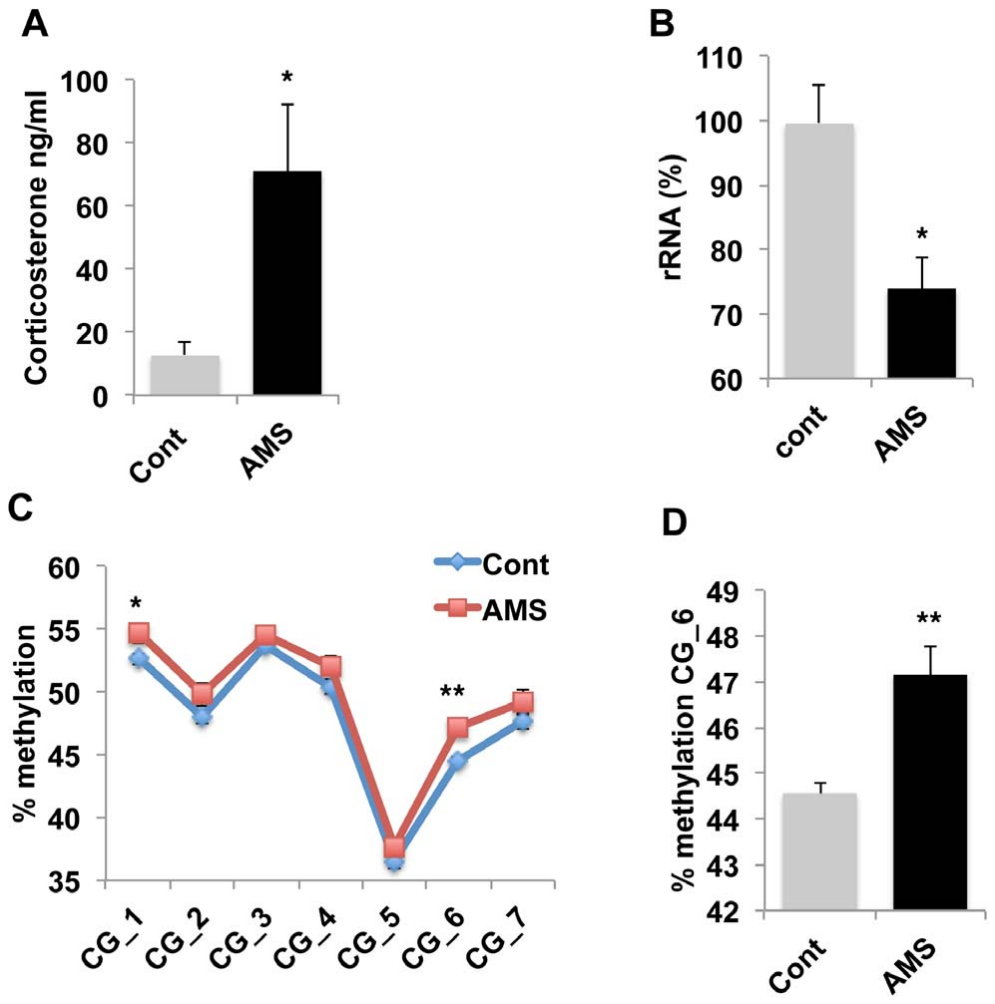
Exposure to 3 hr acute maternal separation (AMS) in 14-day old pups reduces rRNA levels and increases DNA methylation at CG_6. Three hours of maternal separation in 14-day old pups increased plasma levels of corticosterone (A), reduced rRNA levels in the hippocampus (B), and increased DNA methylation at the rDNA promoter (C), most notably CG_6 site (D). Unpaired Student *t* tests (A, B, D), Repeated measures ANOVA followed by simple-effect analysis (C). Error bars represent mean ± SEM, *p<0.05, **p<0.01.

Given that repeated exposure to stress reduces rRNA levels in the developing hippocampus, we wanted to know whether a single acute exposure to maternal separation is sufficient to regulate rRNA availability in the developing hippocampus. To accomplish this, we used a split-litter paradigm in which litters were raised under control condition until PND14, at which point half of the pups were separated from the dam while the other half were left undisturbed in their home cage. After 3 hrs, male pups were processed to assess levels of plasma corticosterone, rRNA, and DNA methylation at the rDNA promoter in the hippocampus. Acute maternal separation (AMS) caused a significant increase in corticosterone levels, t(12) = 2.694, p =  0.02, Fig. 6A, and decreased rRNA levels in the hippocampus, t(11) =  2.50, p =  0.029, Fig. 6B. Repeated measure ANOVA showed an increase in rDNA methylation levels at the rDNA promoter [main effect of AMS, F(1,17) =  6.203, p =  0.023, Fig. 6C], and planned post-hoc analysis confirmed a significant increase in methylation rate at CG_6, t(18) = 3.50. p =  0.003, Fig. 6D. Methylation levels at CG_6 were correlated with corticosterone levels (r =  0.49 p = 0.038), suggesting that elevated corticosterone levels may rapidly regulate DNA methylation at this site. AMS did not affect DNA levels (t(14) = 0.573, p =  0.545) indicating that changes in rRNA levels and DNA methylation occurred in the absence of substantial DNA replication.

## Discussion

A growing body of research has recently demonstrated abnormal hippocampal development in children exposed to early adversity [1]–[5]. These findings challenge earlier assertions in the field suggesting that the effects of childhood maltreatment on hippocampal function are not seen in children, but rather manifest themselves later in adulthood [24]. ELS modifies hippocampal development across diverse mammalian species, including rodents [8], [25], [26], suggesting that animal models may help elucidate the molecular and cellular changes that guide these developmental changes in children. Unfortunately, most of the animal work to date has focused on the effects of ELS on hippocampal function and structure in adulthood, with little effort made to systematically study its effects on neurodevelopment [6], [8]. To address this issue, we developed a robust mouse-model of ELS. This paradigm exposes pups of the highly stress reactive strain, BALB/cByj, to brief daily separation (BDS) during the first three weeks of life in the absence of nesting material (impoverished conditions). Exposure to BDS causes prolonged elevation of corticosterone during the first three weeks of life, blunted hippocampal growth during the second week of life, reduced synaptogenesis (Wei et al. 2014, submitted), and impaired hippocampal-dependent memory in adulthood [8], [9].

Here we tested the hypothesis that BDS impairs hippocampal growth by inhibiting rRNA expression during a critical period of accelerated growth in the developing hippocampus. In support of this hypothesis, we show that the hippocampus undergoes a rapid increase in DNA and RNA content during the second week of life, a process that is accompanied by an increase in the rate of rRNA synthesis. Increased rRNA synthesis during the second week of life was accompanied by a decrease in DNA methylation at rRNA promoter sequences that have been shown to regulate the ability of Pol I to transcribe rRNA [14]. These findings suggest that decreased DNA methylation at the rDNA promoter during the second week supports the accelerated growth by increasing the availability of rRNA. This notion is further supported by the observation that exposure to BDS increases DNA methylation at the rDNA promoter, inhibits rRNA synthesis, and decreases DNA and RNA content in the hippocampus of 14-day old pups.

DNA methylation at the rDNA promoter and rRNA levels were similar in 28-day old control and BDS offspring, suggesting that these processes are not maintained in the absence of ongoing stress. Nevertheless, DNA content in the hippocampus of 28-day old juvenile mice was lower in BDS mice compared to controls, raising the possibility that decreasing rRNA availability at PND14 inhibits DNA synthesis in a manner that persists in the absence of ongoing stress. This assertion is supported by previous work showing that a transient inhibition of protein synthesis in 7-day old rat pups inhibited DNA synthesis, and was associated with reduced DNA content in several brain regions that persisted into adulthood [27], [28]. Moreover, inhibition of rRNA in proliferating NSC caused cell-cycle arrest and apoptosis by activating P53 [12]. Our findings that exposure to acute stress causes a rapid decrease in rRNA levels and an increase in DNA methylation at the rDNA promoter without modifying DNA levels, suggest that transcriptional regulation of rRNA precedes cell cycle arrest in the developing hippocampus. Given that large numbers of NSC proliferate in the hippocampus during the second week of life [13], [29], these findings provide a possible mechanism to explain how BDS regulates neurogenesis and reduces DNA content in the developing hippocampus.

We suspect that the relatively small changes in rRNA levels and DNA methylation seen in BDS mice are due to the cellular heterogeneity that characterizes hippocampal tissue. Additional work is therefore needed to assess the effect of BDS on rRNA levels in more homogenous cell populations (e.g. NSC, pyramidal neurons, and oligodendrocytes) harvested from the developing hippocampus. We believe that such analysis will reveal a much larger effect size of BDS on rRNA levels and DNA methylation. Decreasing rRNA levels is likely to cause different consequences in different cell populations. For example, blocking rRNA synthesis causes rapid cell cycle arrest and apoptosis in NSC, but not in mature pyramidal neurons [12]. It will therefore be interesting to determine whether inhibiting rRNA inhibits synaptogenesis in pyramidal neurons versus myelination in developing oligodendrocytes, processes that are also impaired in the developing hippocampus of BDS mice (Wei et al. 2014, submitted). Finally, exposure to BDS causes a small (10%) but significant decrease in total body weight that persists up to PND28 (data not shown). It will therefore be interesting to test whether BDS decreases rRNA levels while increasing DNA methylation at the rDNA promoter in peripheral tissue as well.

Our work indicates that DNA methylation at CG_6 plays a critical role in regulating the rate of rRNA synthesis in the developing hippocampus. This methylation site is located within the upstream core element (UCE), which is the binding site for the UBF transcription factor. Binding of UBF to this regulatory sequence is required for efficient Pol I transcription of rRNA [30]. The methylation rate at this site is highly correlated with levels of rRNA (Fig. 4C) and are modified in response to age (Fig. 2D), chronic stress (Fig. 3E, 4B), and acute stress (Fig. 6D). These findings are inconsistent with elegant work showing that methylation at the CG_7 site (aka -133), and not CG_6 (aka −143), regulates UBF binding to the UCE and rRNA expression [14]. These divergent outcomes might be due to important differences between the two experimental conditions. For example, we used genomic DNA harvested from the developing hippocampus as opposed to methylated plasmids transfected into NIH3T3 cells (i.e. fibroblasts). It is therefore possible that methylation at the CG_6 site may affect binding of UBF to the UCE in developing neurons exposed to high corticosterone levels (stress). We have attempted to address this issue by using a chromatin immunoprecipitation (ChiP) assay with UBF antibodies. However, we found that the in-vivo UBF-ChiP assay we used was not sufficiently quantitative to address this issue. It is also possible that methylation at CG_6 inhibits rRNA transcription by recruiting a methyl binding transcriptional repressor to the UCE [31]. Finally, we note that acute and chronic early life stress may inhibit rRNA levels by other mechanisms such as phosphorylation of key components of the pol I transcriptional machinery or by modifying rRNA stability [10].

## Conclusions

This work makes several important contributions to previous work examining the mechanisms by which ELS modifies hippocampus development. First, we show that levels of DNA methylation at the rDNA promoter reach a developmental nadir while rRNA levels peak during the second week of life when the hippocampus undergoes accelerated growth. Second, this is the first study to demonstrate that exposure to early life stress increases DNA methylation at the rDNA promoter and reduces rRNA synthesis in the developing hippocampus. Given the critical role that rRNA plays in supporting growth and development, these findings suggest a novel mechanism by which stress early in life impairs hippocampal development. Third, we found that exposure to acute stress early in life is sufficient to reduce rRNA levels and increase DNA methylation at the rDNA promoter, suggesting that stress early in life is able to rapidly regulate rRNA availability in the hippocampus.
